# Prevalence and risk factors analysis for low back pain among occupational groups in key industries of China

**DOI:** 10.1186/s12889-022-13730-8

**Published:** 2022-08-05

**Authors:** Ning Jia, Meibian Zhang, Huadong Zhang, Ruijie Ling, Yimin Liu, Gang Li, Yan Yin, Hua Shao, Hengdong Zhang, Bing Qiu, Dongxia Li, Dayu Wang, Qiang Zeng, Rugang Wang, Jianchao Chen, Danying Zhang, Liangying Mei, Xinglin Fang, Yongquan Liu, Jixiang Liu, Chengyun Zhang, Tianlai Li, Qing Xu, Ying Qu, Xueyan Zhang, Xin Sun, Zhongxu Wang

**Affiliations:** 1 National Institute of Occupational Health and Poison Control, Chinese Center for Disease Control and Prevention, Beijing, China; 2 Chongqing Center for Disease Control and Prevention, Chongqing, China; 3 Hubei Provincial Hospital of Integrated Chinese & Western Medicine, Wuhan, Hubei China; 4 Guangzhou Twelfth People’s Hospital Affiliated to Guangzhou Medical University, Guangzhou, Guangdong China; 5 Liaoning Provincial Health Supervision Center, Shenyang, Liaoning China; 6 Shanghai Center for Disease Control and Prevention, Shanghai, China; 7 Shandong Academy of Occupational Health and Occupational Medicine, Jinan, Shandong China; 8 Jiangsu Provincial Center for Disease Control and Prevention, Nanjing, Jiangsu China; 9 Civil Aviation Medical Center, Civil Aviation Administration of China, Beijing, China; 10 Guizhou Province Occupational Disease Prevention and Control Hospital, Guiyang, Guizhou China; 11 Tianjin Occupational Disease Prevention and Control Hospital, Tianjin, China; 12 Tianjin Center for Disease Control and Prevention, Tianjin, China; 13 Beijing Center for Disease Control and Prevention, Beijing, China; 14 Fujian Province Occupational Disease and Chemical Poisoning Prevention and Control Center, Fuzhou, China; 15 Guangdong Province Hospital for Occupational Disease Prevention and Treatment, Guangzhou, Guangdong China; 16 Hubei Provincial Center for Disease Control and Prevention, Wuhan, Hubei China; 17 Zhejiang Provincial Center for Disease Control and Prevention, Zhejiang, Hangzhou China; 18 Institute of Occupational Medicine of Jiangxi, Nanchang, Jiangxi China; 19 Ningxia Hui Autonomous Region Center for Disease Control and Prevention, Yinchuan, Ningxia China; 20 Sichuan Provincial Center for Disease Control and Prevention, Chengdu, Sichuan China; 21 Shanxi Provincial Center for Disease Control and Prevention, Xian, Shanxi China

**Keywords:** Low back pain Incidence Risk factor

## Abstract

**Background:**

With the acceleration of industrialization and population aging, low back pain (LBP) has become the leading cause of life loss years caused by disability. Thus, it places a huge economic burden on society and is a global public health problem that needs urgent solution. This study aimed to conduct an epidemiological investigation and research on a large sample of workers in key industries in different regions of China, determine the incidence and distribution characteristics of LBP, explore the epidemic law, and provide a reference basis for alleviating global public health problems caused by LBP.

**Methods:**

We adopted a modified epidemiological cross-sectional survey method and a stratified cluster sampling method. All on-duty workers who fulfill the inclusion criteria are taken as the research participants from the representative enterprises in key industries across seven regions: north, east, central, south, southwest, northwest, and northeast China. The Chinese version of the musculoskeletal disease questionnaire, modified by a standardized Nordic questionnaire, was used to collect information, and 57,501 valid questionnaires were received. Descriptive statistics were used, and multivariate logistic regression analysis (*p* < 0.05) was performed to explore the association between musculoskeletal disorders and potential risk factors.

**Results:**

LBP annual incidence among workers in China’s key industries is 16.4%. There was a significant difference in LBP incidence among occupational groups across different industries (*p* < 0.05). The multivariate regression model showed the following as risk factors for LBP: frequent repetitive movements with the trunk, working in the same positions at a high pace, trunk position, frequently turning around with your trunk, often working overtime, lifting heavy loads (i.e., more than 20 kg), education level, staff shortage, working age (years), cigarette smoking, use of vibration tools at work, body mass index, lifting heavy loads (i.e., more than 5 kg), and age (years). Physical exercise, often standing at work, and absolute resting time were protective factors.

**Conclusion:**

LBP incidence among key industries and workers in China is high. Thus, it is urgent to take relevant measures according to the individual, occupational, and psychosocial factors of LBP to reduce the adverse impact of LBP on workers’ health.

## Background

With the development of science and technology and the acceleration of industrialization, significant changes have taken place in the working modes of workers. Workers generally suffer from work-related musculoskeletal diseases (WMSDs) caused by adverse ergonomic factors, such as repetitive operation, bad working posture, excessive force load, continuous muscle tension, and vibration contact. The World Health Organization defines this as “the health problems of muscles, tendons, bones, cartilage, ligaments, nerves, and other motor systems caused or aggravated by work activities, including all forms of health disease states from minor and short-term injuries to irreversible and incapacitated injuries.” Particularly, low back pain (LBP) is the most common condition.

Research shows that [1] about 80% of people globally have experienced LBP. It brings great pain to people, high medical costs, and has a significant impact on the social economy; particularly, the loss of working hours brings a huge medical and economic burden to society [2]. The number of years of disability caused by LBP is estimated to have increased by 54% globally from 1990 to 2015, thus becoming the leading cause of global disability [3]. In March 2018, Lancet published three consecutive reports calling for prompt measures to be taken against the global LBP problem [3–5]. In 2002, the International Labor Organization (ILO) explicitly added musculoskeletal diseases to the International List of Occupational Diseases (recommendation No. 194). Musculoskeletal diseases were further refined in the latest version of the list of occupational diseases approved by the ILO in 2010 [6]. Moreover, LBP has a high incidence in China, causing great harm and severe economic losses. The disease burden report of China and provincial administrative regions from 1990 to 2016 [7] ranked LBP as the first disease causing the loss of life years caused by disability from 2005 to 2016. Therefore, LBP is a global public health problem that needs urgent solution.

Therefore, this study explored the incidence and distribution characteristics of LBP by conducting a large-sample epidemiological investigation and study of key industries in different regions of China, and providing a reference for reducing global public health problems caused by LBP incidence.

## Methods

### Source and study population

This study covers seven regions: north, east, central, south, southwest, northwest, and northeast China. The selection of key industries is based on the representative industries closely related to WMSDs and mentioned in the previous literature, including automobile manufacturing, shoemaking, biopharmaceutical manufacturing, electronic equipment manufacturing, ship and related equipment manufacturing, petrochemical industry, construction, furniture manufacturing, coal mining and washing industry, animal husbandry, medical personnel, automobile 4S shops, vegetable greenhouses, civil aviation crews, and toy manufacturing; a total of 15 industries or working groups. On the one hand, inclusion criteria of the study population were based on workers who are over 18 years old, have worked for more than 1 year, have certain reading and writing abilities, and can understand the meaning of the questionnaire in Chinese. On the other hand, exclusion criteria consisted of people with congenital spinal malformations and patients without WMSDs, such as trauma, infectious diseases, and malignancy. This study has passed the ethical review of the Ethics Review Committee of The Chinese Center for Disease Control and Prevention, and the informed consent was obtained from the participants. Moreover, all methods were performed per relevant guidelines and regulations. Data handling and storage are compatible with this law. All protocols were performed under the Declaration of Helsinki.

### Sample size determination and sampling procedures

This study adopted a stratified cluster sampling method to select all on-duty workers meeting the inclusion criteria from representative enterprises in key industries in north, east, central, south, southwest, northwest, and northeast China. A total of 64,052 people were surveyed and 61,034 questionnaires were received, with a response rate of 94.6% (***95% CI***: 0.951, 0.955); a total of 57,501 valid questionnaires were collected, with an effective rate of 94.2% (***95% CI***: 0.940, 0.944).

### Data collection tool and procedure

The incidence of WMSDs among occupational groups in key industries in different regions of China was investigated using the ergonomic evaluation and analysis system developed by the National Institute for Occupational Health and Poison Control, Chinese Center for Disease Control and Prevention. The system includes four functions: electronic remote operation site ergonomics survey and evaluation tool, real-time data monitoring system, data transmission network, and background data terminal. The survey tool used in this survey is a built-in questionnaire in the system, namely the electronic questionnaire system of the Chinese version of the musculoskeletal disorders questionnaire, which is based on the Nordic Musculoskeletal Disorders Questionnaire (NMQ) [8]. After appropriate modification, it has been proven to have good reliability and validity, and can be used in the Chinese occupational population. The survey contents include ① general information such as age and years of service; ② occurrence of musculoskeletal symptoms; ③ work type, organized form of work, and working posture.

**Fig. 1:**
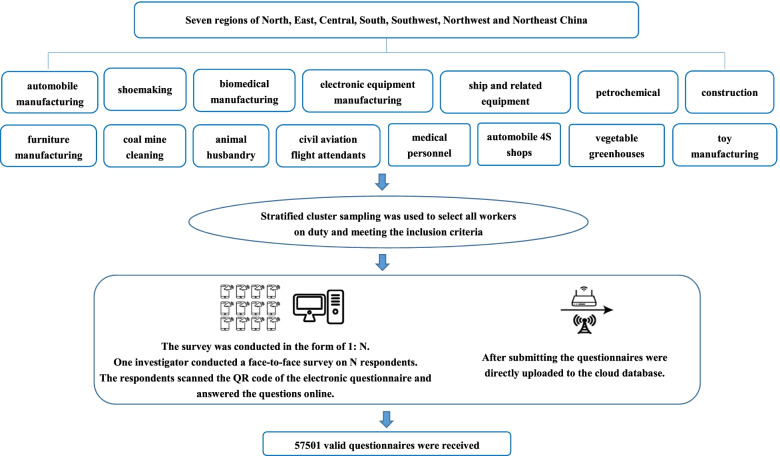
Implementation process of the study

The survey was conducted in 1 an N. One investigator conducted a face-to-face survey with N respondents. The respondents scanned the QR code of the electronic questionnaire and answered questions online. After submitting the questionnaires, they were uploaded directly to the cloud database. Figure 1 shows the implementation process of the study.

### Criteria for LBP

The NIOSH criteria for musculoskeletal injury [9] were adopted: pain, stiffness, burning, numbness or tingling, and other uncomfortable symptoms, which were consistent with ①discomfort in the past year; ② discomfort after accepting the current job; ③ no previous accidents or sudden injuries (local effects and discomfort); ④ if discomfort occurred every month or lasted for more than 1 week, it was judged as a musculoskeletal disease in this part.

### Data quality control

Quality control is conducted throughout the entire research process, including design, implementation, data collection, and data collation, to ensure the scientific nature of the research conclusions and the authenticity, validity, and reliability of the data.

### I Research design

Referring to the relevant literature, clarifying the research purpose, investigation methods, and other vital aspects, and taking appropriate measures to control the possible bias in research design.

### II On-site investigation and measurement

Before the investigation, the investigators were strictly trained to fully understand the purpose and significance of the research and master the investigation and monitoring methods. During the survey, the investigators explained the purpose, significance, and requirements and conducted a face-to-face survey. The participants filled in the questionnaire and submitted it on the spot to ensure the authenticity, integrity, and high retrieval rate of information sources.

### III Data collection

Investigators monitored the completion of the questionnaires to ensure that all surveyed information was from the participants themselves. The electronic questionnaire had a logical error correction to avoid unreasonable information. If there were blank items, the questionnaire could not be submitted. Thus, the investigator assisted the participants to fill in the blanks to ensure that the information is complete.

### Data management and analysis

After the survey data were exported from the backend database, they were statistically processed using SPSS 20.0 statistical software. The measurement data adopted $\overline{\mathrm{X}}\pm \mathrm{s}$ indicators, and the single factor analysis of WMSDs adopts the χ ^2^ test method, multivariate analysis was performed using an unconditional logistic regression model.

## Results

### Socio-demographic characteristics of the study population

This study covers seven regions in north, east, central, south, southwest, northwest, and northeast China. It covers 15 industries or operating groups: automobile manufacturing, shoemaking, biopharmaceutical manufacturing, electronic equipment manufacturing, ship and related equipment manufacturing, petrochemical industry, construction, furniture manufacturing, coal mining and washing industry, animal husbandry, medical personnel, automobile 4S stores, vegetable greenhouses, civil aviation crews, and toy manufacturing. The respondents were 57,501, including 37,240 men and 20,261 women. Of the 57,501 respondents, 7376 (12.8%) were in North China; 19,414 (33.8%) in East China; 2287 (4.0%) in Central China; 18,457 (32.1%) in South China; 3565 (6.2%) in Southwest China; 4391 (7.6%) in the Northwest; and 2011 (3.5%) in the Northeast. Among them 37,240 (64.8%) were men and 20,261 (35.2%) were women; male height:171.10 ± 10.34 cm, weight: 67.83 ± 15.98 kg; female height: 159.57 ± 9.74 cm, weight: 57.24 ± 13.72 kg. The age of the total population was 32.32 ± 9.16 years, and the length of service was 7.51 ± 7.19 years. The educational level, marital status, body mass index (BMI), and smoking status of the total population are shown in Table 1.

**Table 1:** Socio-demographic and Personal characteristics of the study participants, China, 2018–2020 (*n* = 57,501)

Variables	Frequency(n)	Percentage (%)
**Gender**		
Men	37,240	64.8
Women	20,261	35.2
**Age (years)**		
<25	12,085	21.0
25-	26,139	45.5
35-	12,301	21.4
45-	5802	10.1
55-	1174	2.0
**Working age (years)**		
<2	16,061	27.9
2-	12,072	21.0
4-	7299	12.7
6-	9717	16.9
8-	12,352	21.5
**Education level**		
Junior high school	15,369	26.7
Senior high school	21,901	38.1
University degree	19,231	33
Graduate degree	1000	1.7
**Marital status**		
Single	20,997	36.5
Married	35,343	61.5
Divorced/Widowed	1161	2.0
**Body mass index (BMI)**		
<18.5	6006	10.4
18.5-	39,328	68.4
25-	12,167	21.2
**Cigarette smoking**		
No	36,527	63.5
Occasionally	10,111	17.6
Frequently	10,863	18.9

### Prevalence of LBP in key industries in China

The annual LBP incidence in key industries and workers in China is 16.4%. There was a statistically significant difference in LBP incidence among workers across different industries (*p* < 0.05). The LBP incidence in various industries from high to low were vegetable greenhouses (32.5%), toy manufacturing (27.3%), animal husbandry (26.0%), medical personnel (25.3%), biopharmaceutical manufacturing (21.8%), civil aviation crews (20.3%), ship and related equipment manufacturing (18.9%), coal mining and washing industry (17.3%), automobile 4S stores (16.9%), automobile manufacturing (16.0%), electronic equipment manufacturing (13.9%), shoemaking (13.3%), construction (12.0%), furniture manufacturing (10.3%), and petrochemical industry (6.7%). Figure 1 shows these details.

### Factors associated with LBP

Univariate analysis showed that among the single factors, women, age, length of service, educational level, BMI, smoking status, and exercise were all related to the occurrence of LBP (*p* < 0.05). LBP is more common in women than in men. In the control group aged < 25 years, the risk of LBP increased with age before 45 years of age, decreased after 45 years, and slightly increased after 55 years of age. The risk of LBP increases with age, education level, and BMI. Occasional smoking and occasional or regular physical exercise may be protective factors for LBP. Among the workplace factors, frequently standing or kneeling at work, lifting heavy objects more than 5 kg to 20 kg, lifting heavy objects more than 20 kg, using vibration tools at work, working in the same postures at a high pace, bending slightly with your trunk, bending heavily with your trunk, frequently turning around with your trunk, always bending and twisting with your trunk, frequent repetitive movements with your trunk, and working in a bent posture for a prolonged time were associated with the occurrence of LBP (*p* < 0.05). Among psychosocial factors, frequent overtime work, staff shortage, and doing the same job almost daily, were associated with LBP (*p* < 0.05). Abundant resting time, deciding on the rest time independently, and working on rotation may be protective factors for LBP (*p* < 0.05). The results are presented in Table 2.

**Table 2:** Univariate analysis of low back pain among occupational groups in key industries in China, 2018–2020

Variables		Low Back Pain		
	Number of workers	Case	Percentage (%)	***COR(95% CI)***
**Individual risk factors**				
Gender				
Men	37,240	5514	14.8	1
Women	20,261	3935	19.4	1.387(1.326–1.451)^*^
Age (years)				
<25	12,085	1462	12.1	1
25-	26,139	4577	17.5	1.542(1.448–1.643) ^*^
35-	12,301	2238	18.2	1.616(1.505–1.735) ^*^
45-	5802	964	16.6	1.448(1.326–1.581) ^*^
55-	1174	208	17.7	1.565(1.334–1.835) ^*^
Working age (years)				
<2	16,061	1886	11.7	1
2-	12,072	1857	15.4	1.366(1.275–1.464) ^*^
4-	7299	1292	17.7	1.617(1.497–1.746) ^*^
6-	9717	1853	19.1	1.771(1.652–1.899) ^*^
8-	12,352	2561	20.7	1.966(1.843–2.098) ^*^
Education level				
Junior high school	15,369	2225	14.5	1
Senior high school	21,901	3399	15.5	1.085(1.024–1.150) ^*^
University degree	19,231	3626	18.9	1.373(1.296–1.454) ^*^
Graduate degree	1000	199	19.9	1.468(1.249–1.725) ^*^
Body mass index (BMI)				
<18.5	6006	908	15.1	1
18.5-	39,328	6414	16.3	1.094(1.015–1.180) ^*^
25-	12,167	2127	17.5	1.189(1.093–1.295) ^*^
Smoking				
No	36,527	6074	16.6	1
Occasionally	10,111	1453	14.4	0.841(0.791–0.895) ^*^
Frequently	10,863	1922	17.7	1.078(1.019–1.140) ^*^
physical exercise				
No	17,947	3375	18.8	1
Occasionally	32,797	5116	15.6	0.798(0.761–0.837) ^*^
Frequently	6757	958	14.2	0.713(0.660–0.771) ^*^
**Workplace risk factor**				
Standing often at work				
No	8758	1284	14.7	1
Yes	48,743	8165	16.8	1.171(1.099–1.248) ^*^
Sitting often at work				
No	25,385	4134	16.3	1
Yes	32,116	5315	16.5	1.019(0.975–1.066)
Squatting or kneeling often at work				
No	33,942	4750	14.0	1
Yes	23,559	4699	19.9	1.531(1.465–1.601) ^*^
Lift heavy loads (more than 5 kg) kg)				
No	21,719	2787	12.8	1
Yes	35,782	6662	18.6	1.554(1.481–1.630) ^*^
Lift heavy loads (more than 20 kg) kg)				
No	33,670	4669	13.9	1
Yes	23,831	4780	20.1	1.558(1.491–1.629) ^*^
Use vibration tools at work				
No	35,673	5267	14.8	1
Yes	21,828	4182	19.2	1.368(1.308–1.431) ^*^
Working in the same postures at a high pace				
No	11,044	970	8.8	1
Yes	46,457	8479	18.3	2.319(2.162–2.487) ^*^
Trunk posture				
Trunk straight	18,566	2001	10.8	1
Bend slightly with your trunk	31,361	5364	17.1	1.708(1.617–1.805) ^*^
Bend heavily with your trunk	7574	2084	27.5	3.142(2.934–3.365) ^*^
Always turn round with your trunk				
No	20,584	2592	12.6	1
Yes	36,917	6857	18.6	1.583(1.508–1.663) ^*^
Always bend and twist with your trunk				
No	33,046	4069	12.3	1
Yes	24,455	5380	22.0	2.009(1.921–2.100) ^*^
Always make the same movements with your trunk				
No	27,365	2882	10.5	1
Yes	30,136	6567	21.8	2.367(2.258–2.482) ^*^
Work in bent posture for a prolonged time				
No	17,966	2777	15.5	1
Yes	39,535	6672	16.9	1.110(1.058–1.165) ^*^
**Work organization factors**				
Often work overtime				
No	24,830	3385	13.6	1
Yes	32,671	6064	18.6	1.444(1.379–1.511) ^*^
Abundant resting time				
No	31,483	6766	21.5	1
Yes	26,018	2683	10.3	0.420(0.400–0.441) ^*^
Decide the rest time independently				
No	47,308	8015	16.9	1
Yes	10,193	1434	14.1	0.803(0.755–0.853) ^*^
Staff shortage				
No	31,477	4123	13.1	1
Yes	26,024	5326	20.5	1.707(1.633–1.785) ^*^
Do the same job almost every day				
No	7009	716	10.2	1
Yes	50,492	8733	17.3	1.838(1.696–1.992) ^*^
Take turns with colleagues to finish the work				
No	28,980	4944	17.1	1
Yes	28,521	4505	15.8	0.912(0.873–0.953) ^*^

*LBP* Low back pain; Crude odds ratio; *CI* confidence interval; ^*^:*P* < 0.05

The multivariate logistic regression model showed that the influencing factors entered in the model are: frequent repetitive movements with the trunk, working in the same situations at a high pace, trunk position, frequently turning around with your trunk, often working overtime, lifting heavy loads (more than 20 kg), education level, staff shortage and working age (years), cigarette smoking, use of vibration tools at work, BMI, lifting heavy loads (more than 5 kg), age (years), physical exercise, often standing at work, and abundant resting time. The last three are protective factors. The results are presented in Table 3.

**Table 3:** Multivariate logistic regression model predicting the risk factors of LBP among occupational groups in key industries in China, 2018–2020

Variable	Coefficient	Waldχ^**2**^	AOR	95% CI	***P*** value
Always make the same movements with your trunk	0.451	254.409	1.57	1.486–1.66	0.000
Working in the same postures at a high pace	0.338	76.079	1.401	1.299–1.512	0.000
Trunk posture	0.285	208.993	1.33	1.28–1.383	0.000
Always turn round with your trunk	0.247	76.57	1.28	1.211–1.353	0.000
Often work overtime	0.173	45.938	1.189	1.131–1.25	0.000
Lift heavy loads (more than 20 kg)	0.154	26.14	1.166	1.099–1.237	0.000
Education level	0.14	80.491	1.15	1.115–1.186	0.000
Staff shortage	0.138	30.886	1.147	1.093–1.205	0.000
Working age (years)	0.116	162.307	1.123	1.103–1.143	0.000
Cigarette smoking	0.113	41.701	1.119	1.082–1.158	0.000
Use vibration tools at work	0.087	11.129	1.091	1.037–1.149	0.001
Body mass index (BMI)	0.082	14.221	1.086	1.04–1.133	0.000
Lift heavy loads (more than 5 kg)	0.075	5.345	1.078	1.011–1.149	0.021
Age (years)	0.032	4.576	1.033	1.003–1.064	0.032
Physical exercise	−0.125	43.315	0.882	0.85–0.916	0.000
Standing often at work	−0.178	22.741	0.837	0.778–0.9	0.000
Abundant resting time	−0.532	390.399	0.587	0.557–0.619	0.000

*AOR* adjusted odds ratio, *CI* confidence interval

## Discussion

This study investigated the epidemiological characteristics of LBP among occupational populations in key industries in China from January 2018 to December 2020, which is the largest population survey on LBP in China so far. It was found that LBP incidence in key industries or workers in China was 16.4%, which was slightly higher than that reported in other studies. According to the 2010 Global LBP disease burden study report [10], the global LBP incidence was estimated to be 9.4%, with the highest in Western Europe (15.0%), followed by North Africa/Middle East (14.8%), and Central and Latin America (6.6%).

This study found that LBP incidence in greenhouse vegetable farmers was higher than that in other industries. Field investigations have shown that greenhouse planting is a challenging task. Greenhouse vegetable farmers work in greenhouses for at least three-quarters of a year. Owing to the greenhouse’s narrow working space, farmers are predominantly in a bad working posture, such as large forward tilt and bending of the back, and kneeling or squatting for a long time. In addition, owing to the low degree of mechanization of greenhouse operations, there are almost no power tools and auxiliary tools to use, resulting in more repetitive operations and heavy physical labor of greenhouse vegetable farmers. These operational characteristics increase the risk of LBP among greenhouse vegetable farmers. It is worth mentioning that the medical personnel in this survey were also found to have high LBP incidence. An increasing number of domestic and international reports have shown that the incidence rate of WMSDs among medical staff is generally high. This finding is consistent with the results of this study. A survey on WMSDs of dentists in Western countries from 2005 to 2017 showed that the incidence of WMSDs was between 10.8–97.9% and the prevalence in most studies was more than 60% [11], which is higher than the survey results of this study. This may be related to the LBP determination method used in this study. The NIOSH judgment method was adopted in this study. The four judgment criteria are stricter than those of the Nordic Questionnaire [8]. Therefore, the prevalence in this survey was slightly lower than that reported in other surveys.

In terms of individual factors, results showed that age, BMI, smoking status, sports, and other factors are closely related to the occurrence of LBP. The incidence of WMSDs increased linearly with age under 45 years. The cumulative effect can explain this result; with increasing age, the musculoskeletal system of the body shows a trend of degradation. The longer the length of service, the longer the exposure to risk factors. Therefore, acute or chronic loads act on the musculoskeletal tissue, resulting in injury accumulation and increased musculoskeletal diseases [12]. After the age of 45, the incidence of WMSDs showed a downward trend. The field survey found that the management of many enterprises will adjust the operation positions of front-line workers according to the age of workers; that is, front-line workers will be adjusted to auxiliary positions with a light load or promoted to management positions such as team leaders. This may also explain the decline in the incidence of WMSDs. This survey found that the risk of LBP increased with increasing BMI. Houda Ben et al. [13] also found that BMI > 25 kg / m^2^ was closely related to LBP occurrence. Dianat et al. [14] have also found that light BMI is a protective factor for LBP. Further, the survey found that occasional smoking and occasional or regular physical exercise were protective factors against LBP. This finding is consistent with the results of previous studies. Regular smoking aggravates LBP. Abdugad et al. [15] found that smoking is a risk factor for LBP. Smoking causes intervertebral disc degeneration by interfering with intervertebral disc metabolism, proteoglycan, and collagen synthesis, which may lead to LBP [16]. Previous studies have shown that [13, 17], a weekly regular physical activity can reduce LBP risk. According to the American Physical Therapy Association guidelines [18], moderate- to high-intensity exercises are recommended for LBP without pain, and low-intensity exercises for LBP with generalized pain. Research shows that [19] moderate physical exercise can enhance muscle strength and endurance, improve cardiovascular function, promote the diffusion of tissue fluid, ensure the absorption of nutrition by bone and muscle tissue, and alleviate muscle fatigue. Therefore, appropriate physical exercise may reduce the risk of LBP.

In terms of workplace factors, adverse posture operation, frequent repetitive movements as the trunk, working in the same posture at a high pace, bending slightly with the trunk, bending heavily with the trunk, frequently turning around with the trunk, and lifting heavy loads are risk factors for LBP, while often standing at work is a protective factor. Moreover, previous studies have shown that [20], stretching/overstretching and repeated bending at work may be risk factors for LBP. Studies have shown that [21] workers who need to repetitively bend at work are 97% more likely to develop LBP than those who do not.

Research shows that [22], long-term continuous poor posture while working can easily cause blood circulation disorder, serious insufficient blood supply in the spine area, and the inability of the muscles and bones to absorb nutrition, which can easily cause muscle tissue ligament strain. LBP can occur when there is a continuous low-load or short-term strong-load impact. Laboratory research shows that [23, 24], there is a positive correlation between heavy physical load and physical exertion. Coenen et al. [25] found that handling more than 25 kg per day could cause the annual incidence rate of LBP to increase by 4.3%.

In terms of psychosocial factors, this study shows that staff shortages and doing the same job almost daily can increase the risk of LBP, and abundant resting time and deciding the rest time independently can reduce the occurrence of LBP, which is consistent with previous research results. Research shows that [26] psychosocial factors play an important role in the development of LBP. High job requirements are closely related to the occurrence of LBP. Frequently working overtime, a fast work pace, and insufficient time to complete work can lead to WMSDs [27]. According to the 2010 National Health Interview Survey [28], female workers work 41–45 hours a week, and male workers work more than 60 hours a week, increasing the risk of LBP. Therefore, ensuring adequate rest time can relax muscle tissue, reduce the pressure on the lumbar intervertebral disc, and prevent the occurrence of LBP. This study shows that autonomous work progress control is a protective factor against LBP. Domestic and international scholars have reported similar results. Werner et al. [29]. found that a lower perceived decision authority (i.e., lack of rules, decision-making, and participation) is related to wrist WMSDs. If workers can decide the pace of their activities, theoretically, they can avoid activities that aggravate their symptoms and thereby allow healing to occur.

### Limitations

Although this study is a large-sample population survey, it clarifies the epidemic characteristics of LBP in key industries in China and the associations between LBP and its risk factors, which provide essential data for the formulation of LBP prevention and control measures. However, this study has some limitations. First, the research participants are from 15 industries or working groups in China, and some key industries related to LBP have not been investigated; therefore, the deduction is limited. Second, due to this study’s cross-sectional design, it is impossible to make causal inferences between risk factors and LBP. Finally, because this study used a questionnaire survey and the time limit of the questionnaire survey was the past year, the resulting reporting bias and recall bias affected the results.

## Conclusion

In summary, the incidence of LBP among occupational groups in key industries in China was slightly higher than that reported in other countries and regions. The risk factors that may lead to LBP mainly include individual factors, such as age, BMI,

smoking status, and sports; workplace factors, such as poor posture while working and lifting heavy loads; and psychosocial factors, that is, staff shortages and monotonous repetitive work almost daily. Because of this, when making the global public health strategy for prevention, treatment, management, and research of LBP, decision makers and employers should consider the individual, workplace, and psychosocial factors mentioned above to make comprehensive ergonomic preventions and interventions.

## Data Availability

All data generated or analyzed during this study are included in this article. All methods were performed in accordance with relevant guidelines and regulations. The data supporting the findings of this study are also available from the corresponding author upon reasonable request.

## Abbreviations

AOR: adjusted odds ratio
BMI: body mass index
CI: confidence interval
COR: Crude odds ratio
LBP: Low back pain
WMSDs: Work-related musculoskeletal disorders
SPSS: Statistical package for social science
